# Phenotypic Variation of Cell Wall Composition and Stem Morphology in Hemp (*Cannabis sativa* L.): Optimization of Methods

**DOI:** 10.3389/fpls.2019.00959

**Published:** 2019-07-25

**Authors:** Jordi Petit, Agata Gulisano, Annemarie Dechesne, Luisa M. Trindade

**Affiliations:** Wageningen UR Plant Breeding, Wageningen University & Research, Wageningen, Netherlands

**Keywords:** phenotyping methods, fiber quality, genetic diversity, hemp, *Cannabis sativa*, cell wall, stem morphology

## Abstract

The growing demands for sustainable fibers have stimulated the study of genetic diversity in the quality of hemp fiber (*Cannabis sativa* L.). Nevertheless, the lack of high-throughput phenotyping methods that are suited for the analysis of hemp fiber, hampers the analysis of many accessions, and consequently the breeding for this complex trait. In the present report, we developed and optimized the throughput of five methods to study the diversity in hemp fiber quality including cell wall extraction, biochemical composition of cell wall polysaccharides, quantification of lignin, quantification of crystalline polysaccharides and morphology of the stems. Six hemp accessions contrasting for cell wall properties were used to assess the throughput and suitability of these methods for genetic studies. The methods presented revealed to be highly repeatable, with low coefficients of variation between technical replicates. With these methods we were able to detect significant phenotypic variation in cell wall composition and stem morphology between the six accessions. In addition, the throughput of the methods has been upgraded to a level that enables their use for phenotyping cell wall traits in breeding programs. The cell wall extraction was optimized to extract enough material for the complete characterization of the cell wall of hemp while reducing the time for the entire analysis. The throughput of the stem morphological analysis was improved by decreasing the timing of fixation, infiltration, and embedding of mature and dry hemp stems. Notwithstanding, our methods already have the potential to phenotype large number of accessions in a relatively short period of time. Our methods will enable exploration of genetic diversity of fiber quality and will contribute to the development of new hemp varieties with advanced quality of fibers.

## Introduction

We are entering a Circular Bioeconomy era and that requires crops that stand for alternative sustainable solutions. Hemp (*Cannabis sativa* L.) is an environmental friendly and multipurpose crop and therefore it presents an attractive candidate for the bio-based economy (Salentijn et al., 2015). Characterization of hemp fiber quality, including stem morphology and cell wall composition and structure, is the first step toward breeding for high yielding varieties with better fiber quality. Such varieties, that are more amenable to processing, positions hemp as a competitive alternative to poor sustainable fiber crops such as cotton (Ebskamp, 2002; van der Werf and Turunen, 2008; Amaducci and Gusovius, 2010).

Hemp stems are organized in two types of fibers: bast fiber and woody hemp core (WHC) (Crônier et al., 2005). Bast fibers are collections of one to four dozen phloem cells, known as elementary fibers (Thygesen et al., 2006) while WHC fibers are composed of individual xylem cells (Hughes, 2012). The composition of the cell wall is different in the two types of fibers and varies throughout the different developmental stages (Crônier et al., 2005). Despite the differences, both bast and WHC fibers follow a pattern common in dicot plants (Sarkar et al., 2009). Hemp cells from the bast and WHC fibers are surrounded by a middle lamella, a primary cell wall and a secondary cell wall. Furthermore, cells from the bast fiber also have an additional layer, referred to as gelatinous or G-layer (Mellerowicz and Gorshkova, 2012; Behr et al., 2016). The middle lamella is a network mainly composed of pectin and lignin (Willats et al., 2001). The primary cell wall and the secondary cell wall comprised similar components: mainly cellulose, lignin and a matrix of hemicellulosic polysaccharides and pectins (Morrison, 1988; Bonatti et al., 2004), though in different proportions. The G-layer is characterized by a large content of crystalline cellulose and by the presence of rhamnogalacturonan type I (RGI) (Chernova et al., 2018). Cell walls from bast fibers are characterized by a high content of cellulose whereas cell walls from WHC have a larger proportion of lignin and xylans (Bonatti et al., 2004; van den Broeck et al., 2008). In addition, hemp stems are also highly dynamic and they undergo massive modifications during plant development. For instance, lignification of fibers intensifies with flowering and fibers become stiffer (Crônier et al., 2005).

Despite the increasing knowledge on the basics of cell wall composition, little is known about the genetics underlying cell wall biosynthesis and stem morphology in hemp. The complex structure of the cell walls and the organization of different types of cells in the stem limit the development of high-throughput phenotyping methods for the analysis of these traits. In addition, the hydrophilic behavior of certain pectic polymers of hemp such as RGI hampers the complete cell wall extraction with the current high-throughput methods, that are based on aqueous detergents (Goering and Van Soest, 1970; Huffman and Caballero, 2003; Pettolino et al., 2012). Extraction methods based on alcohols [alcohol insoluble residue (AIR)] would probably be better suited alternatives (Pettolino et al., 2012). Yet, AIR protocols currently available use low amounts of starting plant material and consequently several parallel extractions are required before cell wall can be characterized. Thus, the throughput of the hemp cell wall extraction is compromised with the current methods.

The first step to characterize cell wall polysaccharides and lignin comprises the hydrolysis of the carbohydrates into composing monosaccharides (Morrison, 1988). Sulfuric acid (H_2_SO_4_) is a commonly used acid that hydrolyses the majority of the cell wall structures, expect lignin (Morrison, 1988; Sluiter et al., 2012). Notwithstanding, the current H_2_SO_4_ methods produce low repeatable data in hemp. The complex stem morphology and the large crystalline polysaccharides from hemp hampers the complete hydrolysis of the cell wall (Crônier et al., 2005; Pu et al., 2013).

The high crystallinity of cellulose and mannan (Millane and Hendrixson, 1994; Crônier et al., 2005) is thought to play an important role in the properties of bast fibers in hemp, such as fiber strength (Marrot et al., 2013; Placet et al., 2014). The current methods to study the crystallinity of cell wall polysaccharides are based on physical methods (Park et al., 2010), involving many handlings, analysis of only few samples simultaneously and consequently limiting the study of many varieties. The throughput of those methods is a major limitation to study the diversity of crystallinity in hemp bast fiber. Biochemical alternatives adapted to hemp features are a possible alternative to increase the throughput of the analysis. Based on its supramolecular structure, consecutive hydrolysis, using different acids from weak [trifluoroacetic acid (TFA)] to strong (acetic acid – CH_3_COOH, nitric acid – HNO_3_ and H_2_SO_4_) release different amounts of glucose and mannose from cellulose and mannan, respectively (Updegraff, 1969; Foster et al., 2010) at different stages of the treatment. The released monosaccharides in each hydrolysis can be used to determine the percentage of crystalline polysaccharide (Foster et al., 2010).

The different types of hemp fibers and the composition of their cell walls are important for both the architecture of the plant and the fiber quality (Crônier et al., 2005). It is therefore of great interest to understand the composition of the different fibers and how they are organized in the stem to understand the relation to their functionalities. The WHC of mature hemp stems is a hard structure and that hampers fixation, infiltration, and embedding of the stem and consequently their morphological analysis (Crônier et al., 2005; Behr et al., 2016). Hence, methods to fixate, infiltrate and embed mature hemp stems are of great interest.

In the present study, we developed and optimized the repeatability and the throughput of five methods to assess the quality of hemp cell walls including cell wall extraction, biochemical composition of cell wall carbohydrates, quantification of lignin, quantification of crystalline polysaccharides and morphological analysis of mature stems. The methods were specially optimized to phenotype large number of hemp varieties and is a first step toward the development of tools to breed for hemp fiber quality.

## Materials and Methods

### Plant Material

Six fiber hemp accessions were used in this study: CRA410, CRA412, CRA416, CRA420, FNPC243, and WU101 (Table 1). Plants were cultivated in Westerlee (Netherlands, 53°N 6°E) from April to September 2013 and harvested at full flowering.

**Table 1:** Six fiber hemp (*Cannabis sativa* L.) accessions used in this study.

MultiHemp code	Accession name/ code	Origin	Accession type	Provider
CRA410	Ermes A	Italy	Fiber	CRA
CRA412	Carmaleonte	Italy	Fiber	CRA
CRA416	Denise	Romania	Fiber	CRA
CRA420	USO 31	Ukraine	Fiber	CRA
FNPC243	A102-111-1	France	Fiber	FNPC
WU101	JSO 16/891229	Russia	Fiber	WUR

*CRA, FNPC, and WUR stand for Research Centre of Industrial Crops, Federation National Producteurs de Chanvre and Wageningen University & Research, respectively.*

### Preparation of Stem Material

Harvested mature stems were naturally dried, in open air under a roof, until the water content was lower than 18% of the total matter. Thereafter, stems were completely dried in the oven at 60°C for 2 days to prevent initiation of stem retting and samples were stored until analysis. Stems needed for cell wall analysis were chopped to 2 cm length pieces using a chopper machine and re-dried in the oven at 60°C for 1.5 h. Subsequently, samples were grinded to 1 mm size using a grinder Peppink 200AN with a 1 mm sifter. Finally, to ensure that cells were completely disrupted, an extra step of grinding was performed using a Retsch Mixer Mill MM400 Retsch at maximum frequency (30 Hz) for 1 min with a 13 ml stainless steel beaker and two 13 mm steel beads. To avoid an excessive heat up of the samples, grinding jars with the samples were cool down with liquid nitrogen before grinding.

For the crystalline polysaccharide analysis, only bast fiber was used. Harvested mature stems for bast fiber analysis were naturally dried and then were retted as described in van den Oever et al. (2003). Briefly, the stems were warm water retted for 3 days and naturally dried in open air. Thereafter, stems were decorticated with a lab-scaled roller-breaker decortication system according to Wang et al. (2018) followed by a hand-removal of the remaining shives (WHC). Finally, bast fiber was chopped and double grinded as described in the previous paragraph.

For the stem morphology analysis, stored dried stems were used to obtain the cross-sections at approximately 70–80 cm from the ground for the further analysis.

### Cell Wall Extraction

Preparation of cell wall fractions and quantification of the cell wall percentage from the biomass were performed based on the AIR method from (Pettolino et al., 2012) with modifications: sample homogenization, initial biomass amount and drying of cell wall fraction. A summary of the modifications and its implications can be found in Table 2.

**Table 2:** Summary of the modifications of the five protocols.

Protocol		Modification	Reason	Improvement
Preparation of stem material	1)	Extra grinding step (Retsch Mixer Mill MM400 Retsch, 30 Hz for 1’).	Increase the homogeneity of the samples.	Ensure a good representation of the sampled tissues. Increase the repeatability of the cell wall composition analysis.

Cell wall extraction (AIR)	1)	Milled biomass was properly mixed using a vortex.		
	2)	Initial biomass amount was scaled up from 10–50 to 1000 mg.	Extract enough cell wall necessary for its complete characterization in a single extraction.	Reduction of the extraction time.
	3)	Drying of the cell wall fraction using a RapidVap Vacuum Dry Evaporation System.	Speed up the evaporation of alcohols remaining in the cell wall fraction after extraction.	

Two step sulfuric acid hydrolysis (Monosaccharide composition)	1)	Cell wall content amount was decreased to 20 ± 1 mg.	Increase the amount of samples that can be analyzed at the same time, without affecting the repeatability.	Increase the throughput of the cell wall composition analysis (60 samples per batch).
	2)	Centrifuge the tubes after adding the cell wall content.	Concentrate the cell wall on the bottom of the tube to ensure complete hydrolysis.	Increase the repeatability of analysis using low amount of starting cell wall.
	3)	Vortexing step after adding the concentrated acid and centrifuge step.	Increase the mixture of acid with the cell wall to increase the homogeneity of the hydrolysis.	Increase the repeatability of the cell wall composition analysis.
		^∗^Small chemically inert stir bar can be added in the tube.		
	4)	Constant shaping at 200 rpm during the first step of the hydrolysis.	Increase the accessibility of the acid to the entire sample increasing the complete degradation of the crystalline structures.	
	5)	Autoclaving procedure following a similar warming up and cooling down between batches.	Perform the same treatment to all samples.	
	6)	HPAEC: isocratic elution of 20 mM NaOAc in 100 mM NaOH (25’) + linear gradient from 60 mM NaOAc in 100 mM NaOH (15’) to 200 mM of NaOAc in 100 NaOH.	So far, xylose and mannose eluted at the same minute using the HPAEC and it was not possible to distinguish between them.	Analysis of most (neutral and acid) monosaccharides independently in a single run, including xylose and mannose and speed up the analysis.

Klason lignin	1)	Combination of lignin and monosaccharide methods in one procedure using 20 ± 1 mg AIR cell wall fraction.	Reduce the amount of cell wall needed for Klason lignin quantification. Increase the amount of samples that can be analyzed without affecting the repeatability.	Increases the throughput of the cell wall composition analysis and decreases the time of preparation.
	2)	Pre-wash glass fiber prefilters with ultrapure water using the vacuum-filtered system and dried for 16 h at 103°C.	The weight of the glass fiber prefilters might decrease with the filtration of the hydrolyzed cell walls. The pre-washing keeps the weight constant. This is especially important when the initial amount of cell wall is really small (≈20 mg).	Increase the repeatability of the analysis.

Crystalline polysaccharides	1)	Cell wall content amount was increased to 20 ± 1 mg.	The original protocol (2 mg) was optimized in Arabidopsis but when applied in hemp the repeatability was low.	
	2)	Third hydrolysis was changed from Saeman hydrolysis (72% H_2_SO_4_) to the two step sulfuric acid hydrolysis.	Hemp has large crystalline fraction reason why this hydrolysis was changed by the two step sulfuric acid hydrolysis (72% + 4% H_2_SO_4_).	Ensure the complete hydrolysis of highly crystalline structures and increase the repeatability of the analysis.

Stem morphology	1)	Fixation was changed from 72 to 48 h.	Long steps that can be reduced without affecting the process.	Reduction of preparation time. Complete preparation of samples in 4 days while the original protocol was 7 days.
	2)	Each step of the dehydration was changed from 2 h to 30 min.		
	3)	First step of the infiltration was changed from overnight to 2 h.		

Milled plant material was mixed using a vortex to ensure a good representation of the sampled tissues, especially for the stem samples. From these samples the AIR fraction was extracted, corresponding to the total cell wall fraction, and used as starting material for the characterization of the cell wall, including monosaccharide composition, quantification of lignin and crystalline polysaccharide analysis. The protocol was scaled up to 1 g starting material and the extraction was performed in 50 ml disposable Nunc tubes suitable for ultracentrifugation. The AIR extraction consisted of two steps: extraction of cell wall and α-amylase digestion.

Cell wall extraction was performed with 36 ml of 80% ethanol (vol/vol) for 30 min on ice. Samples were properly mixed using a vortex after the ethanol was added and every 10 min. Cell walls were ultracentrifuged for 5 min at 10,000 *g* at 4°C using a ultracentrifuge Beckman Avanti with a Fibrelite R14BA – 14x50cy rotor and the supernatant discarded. This extraction step was repeated three times. Lipids and chlorophyll were removed with 36 ml of absolute acetone at room temperature for 10 min. After adding the acetone, samples were mixed using a vortex. Thereafter, samples were ultracentrifuged during 5 min (10,000 *g*, at room temperature) and the supernatant discarded. The remaining chlorophyll was removed with 36 ml of absolute methanol at room temperature. After a vortexing step, the extraction proceeded for 10 min and samples were ultracentrifuged again (5 min, at 10,000 *g*, at room temperature) and the supernatant discarded. Finally, the pellet was dried using a RapidVap Vaccum Dry Evaporation System (Labconco, Kansas City, MI, United States) decreasing gradually the pressure with constant shaking.

Extracted cell walls were incubated with α-amylase (porcine pancreas, Megazyme) to remove starch from the AIR fraction. Dried samples were incubated in 6 ml of 10 mM Tris-maleate (Pettolino et al., 2012) buffer for 30 min with constant shaking. Starch was gelatinized by boiling the samples in a pot with boiling water for 5 min followed by a cool down of the samples to 40°C with ice. Thereafter, two rounds of α-amylase digestion were conducted. The first one consisted of 2 U of enzyme for mg of carbohydrate diluted in 2 ml of 10 mM Tris-maleate buffer for 1 h at 40°C in a HLC thermomixer with smooth shaking (300 rpm). The second digestion consisted of half the amount of enzyme for 30 min, under the same conditions as the first digestion (40°C and 300 rpm).

After the digestion, α-amylase was inactivated by adding 36 ml of cold absolute ethanol and precipitated at -20°C for 1 h, with a previous vortexing step. Samples were centrifuged in a Multifuge 3S Heraeus with a Sorvall Heraeus rotor for 5 min at 1,500 *g* at room temperature. Supernatant was discarded and three extra washes of 36 ml of absolute ethanol with a vortexing step and 5 min centrifuge (1,500 *g*, room temperature) in between were performed. Finally, pure AIR fraction was dried using a RapidVap system as described in the previous paragraph of this section.

Quantification of the cell wall percentage was performed following the original protocol (Pettolino et al., 2012) (50 mg as initial weight in a 2 ml microcentrifuge tube) in triplicates with a single modification. The percentage of cell wall was calculated as the difference between the initial weight of the milled plant material and the weight of the sample after the extraction, corrected for dry matter content.

### Monosaccharide Composition

The AIR fraction was hydrolyzed to analyze the biochemical composition of the cell wall. A two-step sulfuric acid hydrolysis, 72% H_2_SO_4_ (w/w) at 30°C followed by 4% H_2_SO_4_ (w/w) at 121°C was used to hydrolyze the polysaccharides. The protocol used was based on the one described by Sluiter et al. (2012) with modifications (Table 2).

Twenty mg of AIR fraction were weighted into a 12 ml glass tube (Schott^®^ culture tube: 18 mm of diameter and 113 mm of length) with a screw leak-proof cap (Schott^®^ red screw cap: DIN thread GL 23 mm of diameter and 20 mm of height) suitable for the autoclave. Tubes were centrifuged for 1 min at maximum speed in a Multifuge 3S Heraeus with a Sorvall Heraeus rotor to concentrate the AIR fraction at the bottom of the tube. A volume of 0.4 ml of 72% H_2_SO_4_ was added in the tube and mixed using a vortex. When the AIR fraction was difficult to suspend in the acid, a small chemically inert stir bar was put in the tube and vortexed, followed by a spin down in the centrifuge to concentrate the cell walls and the acid. The stir bar was left in the tube for the entire procedure. The tubes were placed in a New Brunswick Scientific INNOVA42 incubator at 30°C for 1 h with constant shaking at 200 rpm. After the first hydrolysis, acid was diluted to 4% by adding 11.42 ml of ultrapure water (milli-Q^®^). Tubes were properly capped, vortexed, and autoclaved (Tuttnauer autoclave 3850EL – D, Breda, Netherlands) at 121°C for 1 h. The autoclaving step followed a similar warming up and cooling down procedure between batches to ensure repeatability between them. Upon hydrolysis, samples were centrifuged at 3,500 *g* at room temperature for 10 min. After the second hydrolysis, 2 ml of supernatant were filtered using 0.45 μm PTFE membrane filters and 1x and 10x dilutions of the filtered material were prepared for carbohydrate analyses.

A set of sugar recovery standards (SRSs) was prepared to quantify the amount of monosaccharides degraded in the second hydrolysis step and to use them as correction factors. SRS solution included D-(-)arabinose, D-(+)galactose, D-(+)galacturonic acid, D-(+)glucose, D-glucuronic acid, D-(+)mannose, L-(+)rhamnose and D-(+)xylose in ultrapure water, in a specific concentration for each monosaccharide that resembles the concentration in the hemp cell wall (Supplementary Table 1). Two control SRS tubes (no hydrolysis) were prepared adding 11.42 ml of SRS solution with 0.4 ml of ultrapure water and two hydrolysis SRS tubes were prepared adding 0.4 ml of 72% H_2_SO_4_ instead of ultrapure water. The two hydrolysis SRS tubes were hydrolyzed in the same way as the cell wall samples. Finally, all SRS were filtered and diluted 1x and 10x as the hemp samples and they were analyzed as follows.

Monosaccharide composition of the cell wall hydrolysates and SRS were analyzed using high performance anion exchange chromatography (HPAEC) on a Dionex^TM^ ICS-5000^+^ DC equipped with a Dionex CarboPac^TM^ PA-100G BioLC^TM^ column (2 mm × 250 mm) preceded by a similar guard column (2 mm × 250 mm) (Thermo Fisher Scientific, Sunnyvale, CA, United States). Separation of monosaccharides was performed at a flow rate of 0.25 ml/min at 30°C. To quantify arabinose, galactose, galacturonic acid, glucuronic acid, mannose, rhamnose, and xylose 5 μl of 1x diluted samples were injected in the column using a Dionex AS-AP auto-sampler and to quantify glucose, only 2.5 μl of 10x diluted samples were injected. The separation method consisted of an isocratic elution of 20 mM sodium hydroxide (NaOH) for 25 min followed by a linear gradient starting at 60 mM sodium acetate (NaOAc) in 100 mM NaOH for 15 min and ending at 200 mM of NaOAc in 100 mM NaOH. The column was washed with 1M NaOAc in 100 mM NaOH for 5 min prior to re-equilibration for 30 min in 20 mM NaOH. Finally, the eluent was monitored by a 30°C thermostatted Thermo Scientific ICS-5000^+^ pulsed electrochemical detector (PAD) (Thermo Fisher Scientific, Sunnyvale, CA, United States).

The contents of monosaccharides detected by HPAEC-PAD were corrected for the percentage of monosaccharides degraded during the second step hydrolysis derived from the SRSs. The percentage of each monosaccharide was calculated relatively to the initial amount of cell wall (AIR fraction) and the cell wall percentage. Monosaccharide composition was analyzed in triplicate in different hydrolysis batches.

### Quantification of Lignin

The content of lignin was analyzed based on an adapted Klason lignin (KL) method essentially as described by van der Weijde et al. (2016) with modifications (Table 2). The initial material consisted of AIR fraction instead of neutral detergent fiber (NDF) (Goering and Van Soest, 1970). KL consisted of the insoluble fraction of the cell wall to H_2_SO_4_ after the two step-hydrolysis, previously described in Section “Monosaccharide Composition”; which is considered to be mainly lignin (Sluiter et al., 2012). After the hydrolysis, samples were cooled down and vacuum-filtered using a glass filtering crucible (30 ml, P4, Klaus Hoffmann, Staudt, Germany) with a pre-washed glass fiber prefilter (EMD Millipore^TM^ AP4004700, MERK) to collect the KL fraction. The residues were dried for 16 h in the oven at 103°C and weighed to calculate the percentage Klason lignin in the AIR fraction. The analysis was performed in triplicate. A crucial step to increase the repeatability of the analysis was the pre-wash of the glass fiber pre-filters with ultrapure water using the vacuum-filtered system and weighted them after being dried for 16 h in the oven at 103°C.

### Analysis of the Crystallinity of Cell Wall Polysaccharides

The percentage of crystalline polysaccharides in the bast fiber was analyzed based on an adapted Updegraff method (Foster et al., 2010) with modifications (Table 2). The method consists of three consecutive hydrolysis with several washing steps in between to remove the acid and released monosaccharides. The first hydrolysis uses a weak acid, TFA, that targets mainly non-crystalline polysaccharides such as xylans and pectins and amorphous cellulose and mannan. The second hydrolysis uses the Updegraff solution, a combination of acetic acid, nitric acid, and water in a 8:1:2 ratio (Updegraff, 1969). Updegraff hydrolysis weakens the crystalline structure of cellulose and mannose to assist the next hydrolysis. The third hydrolysis uses a strong acid (H_2_SO_4_) to break down the crystalline polysaccharides (Foster et al., 2010) uses Seaman hydrolysis consisting of a single step hydrolysis with 72% H_2_SO_4_. Yet, we exchanged this hydrolysis for the two-step H_2_SO_4_ hydrolysis described in Section “Monosaccharide Composition” to ensure the complete hydrolysis of the highly crystalline structures of hemp.

The amount of starting material was scaled up to twenty mg of AIR fraction prepared from the bast fiber to increase the repeatability of the analysis. AIR fraction was weighted into a 12 ml glass tube (Schott^®^ culture tube: 18 mm of diameter and 113 mm of length) with a screw leak-proof cap (Schott^®^ red screw cap: DIN thread GL 23 mm of diameter and 20 mm of height) suitable for the autoclave. Tubes were centrifuged for 1 min at maximum speed. A volume of 2.5 ml of 2M TFA was added to the tubes and incubated for 90 min at 121°C in a HLC Heating thermomixer with smooth shaking. After the first hydrolysis, tubes were cooled down on ice and centrifuged at 3,500 *g* at room temperature for 30 min using a Multifuge 3S Heraeus with a Sorvall Heraeus rotor. Two ml of supernatant were collected, diluted 10x and stored for further analysis. The remaining TFA and released monosaccharides were washed out with 3 ml of isopropanol. Tubes were centrifuged at 3,500 *g* at room temperature for 30 min and supernatant discarded but approximately 1.5 ml was left in the tube to avoid disturbing the pellet. The washing step was performed three times. To dry the pellets, tubes were placed in a HLC thermomixer at 40°C with smooth shaking and a constant stream of nitrogen gas was injected inside the tubes.

The second hydrolysis consisted of 10 ml of Updegraff solution at 100°C in a HLC thermomixer for 30 min. Tubes were centrifuged at 3,500 *g* at room temperature for 50 min and supernatant discarded leaving approximately 1.5 ml of supernatant. Updegraff solution was washed out with 10 ml of ultrapure water, tubes were shake, centrifuged (3,500 *g*, for 50 min, at room temperature) and supernatant discarded. Thereafter, the washing step was repeated three more times with 10 ml of acetone. Finally, pellets were dried in a HLC thermomixer at 40°C with smooth shaking and constant injection of nitrogen gas. The third hydrolysis with two-step H_2_SO_4_ was performed as described in Section “Monosaccharide Composition.”

Monosaccharides released upon TFA and H_2_SO_4_ hydrolysis were determined with a HPAEC-PAD. Total content of cellulose and total content of mannan were the sum of glucose and mannose, respectively released from TFA and H_2_SO_4_ hydrolysis. Content of cellulose and content of mannan in crystalline forms were determined by the content of glucose and the content of mannose, respectively released from only the H_2_SO_4_ hydrolysis. The percentage of crystalline cellulose and mannan were calculated as follows:

(1) ${\text{Percentage of crystalline cellulose, \%Cryst}}_{\text{cell}}=\frac{\text{Content of Cellulose in crystalline form}}{\text{Total content of Cellulose}}*100$
(2) ${\text{Percentage of crystalline mannan, \%Cryst}}_{\text{man}}=\frac{\text{Content of mannan in crystalline form}}{\text{Total content of mannan}}*100$

Analyses were performed in triplicate in different batches.

### Morphological Analysis of Mature Hemp Stems

Dry mature stems were cut in smalls disks (0.5 cm of length) by using a vertical band saw (FERM SSM1005 Scroll Saw – 90W) and to avoid separation between bast and WHC during the cutting process, stems were covered with tissue paper and parafilm^®^ M. Stem sections were fixated, infiltrated and embedded using Technovit^®^ 7100 Kit (Heraeus Kulzer) according to the specifications of the manufacturer with modifications (Table 2), in particular incubation times were optimized. Sections were fixed in 0.1M phosphate buffer with 5% glutaraldehyde under vacuum for 48 h. To remove the fixative, samples were washed under vacuum with 0.1M phosphate buffer (4 min × 15 min) and subsequently with water (2 min × 15 min). Thereafter, stem sections were dehydrated in a series of 10, 30, 50, 70, 96, and 100% ethanol under vacuum for 30 min each dilution. Samples were pre-infiltrated with a solution of 100% ethanol and Technovit A solution (Technovit liquid 100 ml with 1 g of Hardener I) in a ratio 1:1 under vacuum for 2 h. Subsequently, samples were infiltrated with Technovit A solution under vacuum for 24 h. Finally, samples were embedded in the microtome molds with freshly prepared embedding solution (15 ml of Technovit A solution with 1 ml Hardener II). Once the specimens were solidified, slices of 2 to 5 μm of thickness were cut with a rotary microtome (Reichert-Jung 2055).

**FIGURE 1:**
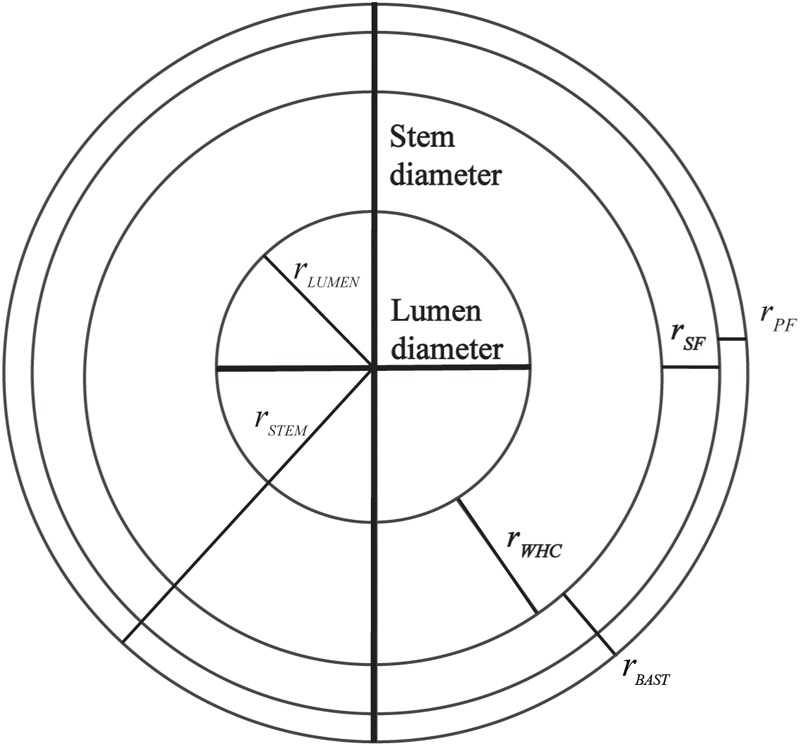
Radius of the different parts of the stem that were used to determine the areas and ratios of different stem structures.

For microscopy observations, slices were stained with Toluidine blue according to O’Brien et al. (1964) with modifications: 0.1% Toluidine blue in 0.1M phosphate buffer. Dense cellulose fractions (bast fiber) are stained in pink and dense lignin fraction (WHC fiber) in blue. Additionally, the detection of the bast and the WHC could also be performed with immunohistochemistry, as Technovit 7100 is compatible with antibodies. Behr and colleagues reported immunohistochemistry in the hypocotyl of hemp using a set of antibodies on stems embedded with Technovit 7100. LM5, LM10, LM15, and CMB3a from Plant Probes are antibodies specific to β-1,4-galactan, xylan, xyloglucan, and crystalline cellulose, respectively. This set of antibodies was used to differentiate the bast from the WHC (Behr et al., 2016). Observations of the Toluidine blue staining were performed with a light microscope (Axiophot Zeiss) connected to an axioCam digital camera and with a handheld digital microscope (Dino-lite) using Dinocapture software. Morphological analysis of stem included measurements of the radius of bast (primary and secondary bast fiber), WHC, lumen and stem diameter which were performed using Gimp2 and ImageJ software. Figure 1 illustrates the measurements of the radius that were taken from each microscopy observations. Measurements were performed in triplicate in each slice and each sample was measured in three slices. The measurements were used to determine the areas and ratios of the different stem structures:

(3) Area of lumen, A_Lumen_ = π ∗ r_Lumen_^2^
(4) Area of WHC, A_WHC_ = [π ∗ (r_Lumen_ + r_WHC_)^2^] - A_Lumen_
(5) Area of bast fiber, A_Bast_ = [π∗(r_Lumen_+r_WHC_+r_Bast_)^2^]-(A_Lumen_+ A_WHC_)

where r_Pith_, r_WHC_, and r_Bast_ are the radius of lumen, WHC and bast, respectively.

(6) $\text{Bast area\% }=\frac{{\text{A}}_{\text{Bast}}}{{\text{A}}_{\text{WHC}}{\text{+A}}_{\text{Bast}}}*100$
(7) $\text{Ratio bast/WHC }=\frac{{\text{A}}_{\text{Bast}}}{{\text{A}}_{\text{WHC}}}*100$
(8) Area of primary bast fiber, A_PF_ = π ∗ ((r_Stem_^2^) - (r_Stem_ - r_PF_)^2^)
(9) Area of secondary bast fiber, A_SF_ = π ∗ ((r_Stem_ - r_PF_)^2^ - (r_Stem_ - (r_PF_ + r_SF_))^2^)

where r_PF_ and r_SF_ are the radius of lumen, primary and secondary bast fiber, respectively.

(10) $\text{Ratio primary bast fiber/secondary bast fiber = }\frac{{\text{A}}_{\text{PF}}}{{\text{A}}_{\text{SF}}}$

### Statistical Analyses

Coefficients of variation (CV%) between technical replicates were used to evaluate the repeatability of the methods:

(11) $\text{Coefficient of variation }=\frac{\text{Standard deviation}}{\text{Mean}}\;*100$

ANOVA was used to determine significant differences between accessions for all the traits analyzed and to detect differences between tissues. Statistical analyses were performed using Genstat 19^th^ edition software (VSN International, Hemel Hempstead, United Kingdom). Correlation analysis between stem morphological parameters and cell wall traits were performed in R version 3.4.3 statistical software using corrplot function.

## Results

### Cell Wall Composition of Hemp: Bast Fiber and Stem

**FIGURE 2:**
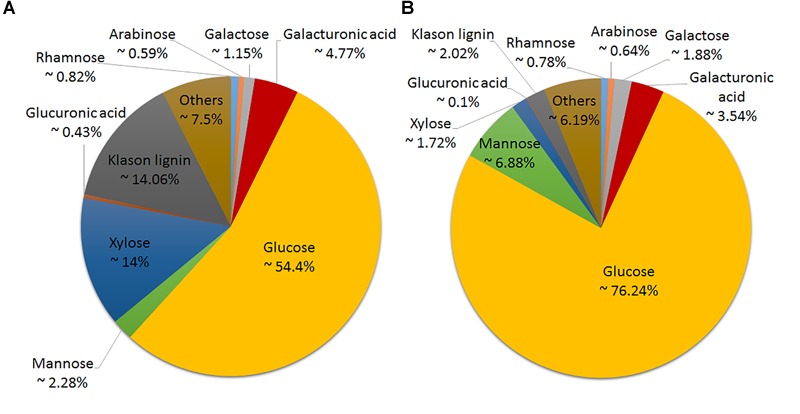
Cell wall composition of hemp stem **(A)** and hemp bast fiber **(B)** as an average of the six hemp accession analyzed.

**FIGURE 3:**
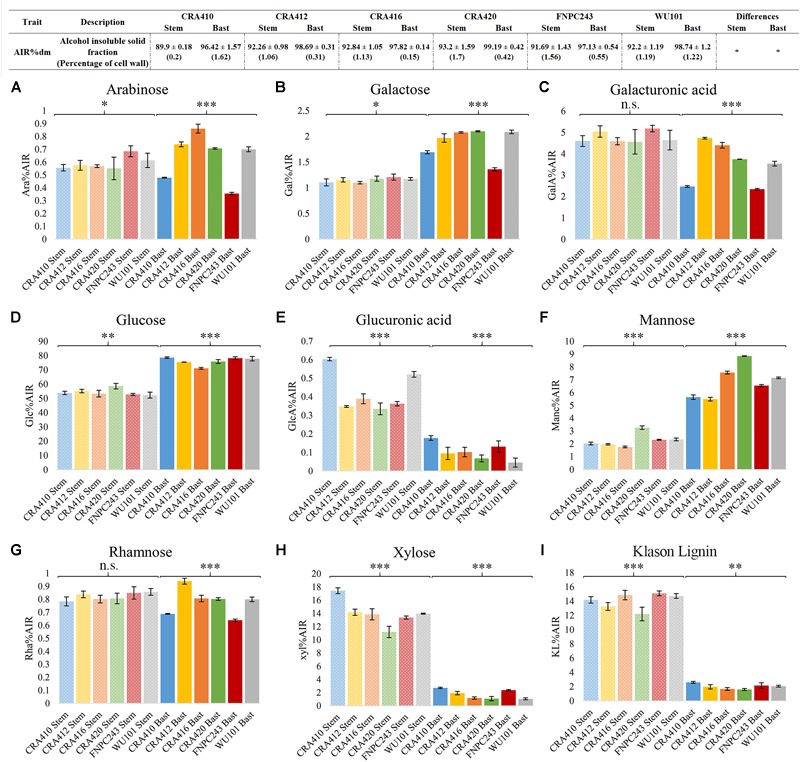
Cell wall composition of the bast fiber and the entire stem of six hemp contrasting accessions. **Table:** The values correspond to the mean ± standard deviation. Percentages of coefficient of variation (CV%) between technical replicates are shown between brackets. **Figure:** The columns represent the means and the bar of each column represents the standard deviation. The contents of the nine cell wall components [arabinose **(A)**, galactose **(B)**, galacturonic acid **(C)**, glucose **(D)**, glucuronic acid **(E)**, mannose **(F)**, rhamnose **(G)**, xylose **(H)**, and Klason lignin **(I)**] were significantly different (*p* < 0.001) between the bast and the stem. ^∗^*p* < 0.1, ^∗∗^*p* < 0.01, ^∗∗∗^*p* < 0.001, and n.s., no-significant.

Six hemp accessions were analyzed for the content and composition of the cell wall in the stem and in the bast fiber. Figure 2 depicts the overall cell wall composition in the stem and bast fiber reported as the average between accessions. The bast fiber was composed almost exclusively of cell wall (98% AIR%dm) whereas the percentage of cell wall in the whole stem was 92% (Figure 3). Glucose, mostly released during hydrolysis of cellulose (Crônier et al., 2005), was the main component of the cell wall accounting for 76.24 and 54.4% of the cell wall from the bast and the stem, respectively. The two second main components of the whole stem were xylose, which mainly composes xylan (Pauly et al., 2013), and Klason lignin, accounting for 14 and 14.06% of the total cell wall, respectively. These components only accounted for 1.72 and 2.02% of the bast, respectively. Glucuronic acid, a monosaccharide associated to xylan (Pauly et al., 2013) was also present in higher content in the stem than in the bast: 0.43 versus 0.1%. Mannose, the main component of mannan (Pauly et al., 2013) was present in higher content in the bast fiber than in the stem, 6.88 and 2.28%, respectively. The two monosaccharides in the backbone of pectin –galacturonic acid and rhamnose- (Willats et al., 2001) were found in larger content in the stem than in the bast: 4.77 and 0.82 versus 3.54 and 0.78%. The content of the other pectic monosaccharides –arabinose and galactose- (Willats et al., 2001) was slightly higher in the bast than in the stems (Figure 2, 3). Altogether, the sum of the measured monosaccharides and lignin content reached 93.8% of the total cell wall for the bast and 92.5% for the stem.

### Cell Wall Composition and Content in the Six Hemp Accessions

Phenotyping methods suitable for breeding programs need to be reproducible and high-throughput to distinguish the phenotypes between accessions and to enable analysis of a large number of samples. Six contrasting hemp accessions were used to evaluate the suitability of the optimized methods for breeding for hemp biomass quality. Most parameters showed significant differences between accessions in both tissues, with the exception of cell wall content, as depicted in Figure 3. Contents of glucose, glucuronic acid, mannose, xylose, and Klason lignin showed large and significant differences between accessions in the stems. Xylose showed the largest range of variation between the six accessions, CRA410 showed 17% of xylose in the cell wall while CRA420 showed only 11% of xylose. Arabinose, and galactose showed small but significant differences between accessions while galacturonic acid and rhamnose showed no-significant differences in the stem. In the bast fiber, all cell wall components showed large significant variation and mannose showed the largest range of variation: CRA412 contained 5.5% of mannose in the bast cell wall while for CRA420 it amounted 8.85% (Figure 3 and Supplementary Table 2).

### Most of the Cellulose and Mannan in the Bast Fiber Were in a Crystalline Form

The largest proportion of cellulose composing the bast fiber fraction was present in the crystalline form, on average 94% of the cellulose was crystalline (Table 3 and Supplementary Figure 1). Mannan was also highly crystalline, approximately 65% of the total. Both polysaccharides showed significant differences between accessions regarding the proportion of crystalline structures relatively to the total. The range of variation for crystalline cellulose was 1.81% between the six accessions and the range of crystalline mannan was much larger, 17.10%. CRA420 showed the lowest content of crystalline cellulose and mannan, 93.23 and 56.04% respectively, whereas CRA410 showed the largest percentage of crystalline polysaccharides, 95.05 and 73.13%.

**Table 3:** Percentage of crystalline cellulose and mannan in the bast fiber of six contrasting hemp accession.

Trait	CRA410	CRA412	CRA416	CRA420	FNPC243	WU101	Sign. level
Percentage of Crystalline cellulose (%Cryst_cell)	95.05 ± 0.588 (0.62)	94.50 ± 0.167 (0.18)	93.66 ± 0.586 (0.63)	93.23 ± 0.296 (0.32)	94.56 ± 0.233 (0.25)	94.30 ± 0.181 (0.19)	^∗∗^
Percentage of Crystalline mannan (%Cryst_man)	73.13 ± 2.557 (3.50)	65.09 ± 0.242 (0.37)	61.90 ± 3.425 (5.56)	56.04 ± 3.117 (5.56)	69.54 ± 1.784 (2.57)	62.78 ± 1.788 (2.85)	^∗∗∗^

*Values indicated correspond to mean ± standard deviation. Percentages of coefficient of variation (CV%) between replicates are shown between brackets. ^∗∗^*p* < 0.01 and ^∗∗∗^*p* < 0.001.*

### Galacturonic Acid, Rhamnose, and Xylose Were Also Detected in the Crystalline Fraction of the Bast Fiber

Galacturonic acid, rhamnose, and xylose were also detected after the two-step sulfuric acid hydrolysis of the crystalline cell wall fraction, as shown in Table 4 and in Supplementary Figure 1. On average 0.5% of xylose content was detected in the crystalline fraction, which represented between 24.26 and 39.46% of the total xylose content, depending on the accession. A larger content of galacturonic acid content was detected in the same fraction, 0.85% as an average, which represented between 25.9 and 47.6% of the total galacturonic acid content. Both carbohydrates were significantly different between the six accessions but galacturonic acid showed a larger variation. Xylose content in the crystalline fraction ranged from 0.272 to 0.845% whereas galacturonic acid ranged from 0.366 to 1.485%. Small amounts of rhamnose were also identified in the crystalline section, 0.006% as an average, which only represents between 0.76 and 0.9% of the total rhamnose content. However, no significant differences between accessions were found for rhamnose.

**Table 4:** Content of xylose (Xyl), galacturonic acid (GalA), and rhamnose (Rha) detected in the crystalline fraction of the six hemp accessions.

Trait	CRA410	CRA412	CRA416	CRA420	FNPC243	WU101	Sign. level
Percentage of Xyl in the crystalline fraction (%)	0.780 ± 0.131 (16.92)	0.34 ± 0.04 (24.26)	0.297 ± 0.054 (18.2)	0.272 ± 0.122 (44.83)	0.84 ± 0.21 (25.1)	0.45 ± 0.19 (42.9)	^∗∗∗^
Percentage of Xyl detected to the total Xyl	31.15	12.77	25.6	27.4	35.38	39.46	
Percentage of GalA in the crystalline fraction (%)	0.366 ± 0.036 (9.77)	1.18 ± 0.10 (8.74)	1.485 ± 0.206 (13.87)	0.838 ± 0.083 (9.9)	0.371 ± 0.016 (4.3)	0.868 ± 0.05 (6.22)	^∗∗∗^
Percentage of GalA detected to the total GalA	25.9	40.63	47.6	38.66	26.55	39.86	
Percentage of Rha in the crystalline fraction (%)	0.0053 ± 0.00085 (16.09)	0.0067 ± 0.00055 (8.19)	0.0073 ± 0.00052 (7.13)	0.0056 ± 0.0012 (21.87)	0.0045 ± 0.00043 (9.5)	0.0061 ± 0.0011 (18.01)	n.s.
Percentage of Rha detected to the total Rha (%)	0.76	0.71	0.9	0.7	0.71	0.77	

*Values indicated correspond to mean ± standard deviation. Percentages of coefficient of variation (CV%) between replicates are shown between brackets. ^∗∗∗^*p* < 0.001 and n.s., no-significant respectively.*

### Stem Morphological Measurements Were Significantly Correlated to Most Cell Wall Traits

**FIGURE 4:**
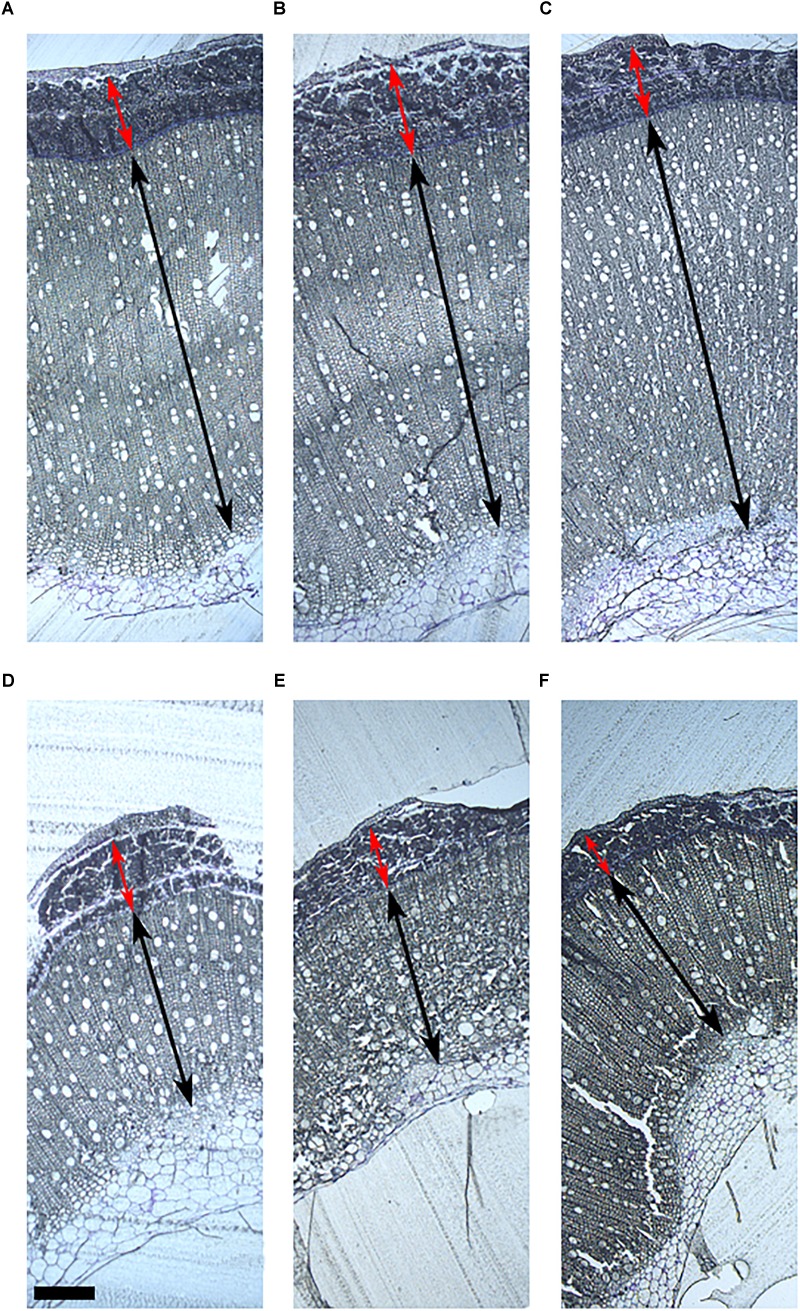
Stem morphology of six contrasting hemp accessions. **(A)** CRA412, **(B)** FNPC243, **(C)** WU101, **(D)** CRA410, **(E)** CRA416, and **(F)** CRA420. Red arrows indicate the bast fiber, black arrows indicate the woody hemp core and scale bars are equivalent to 1000 μm.

**FIGURE 5:**
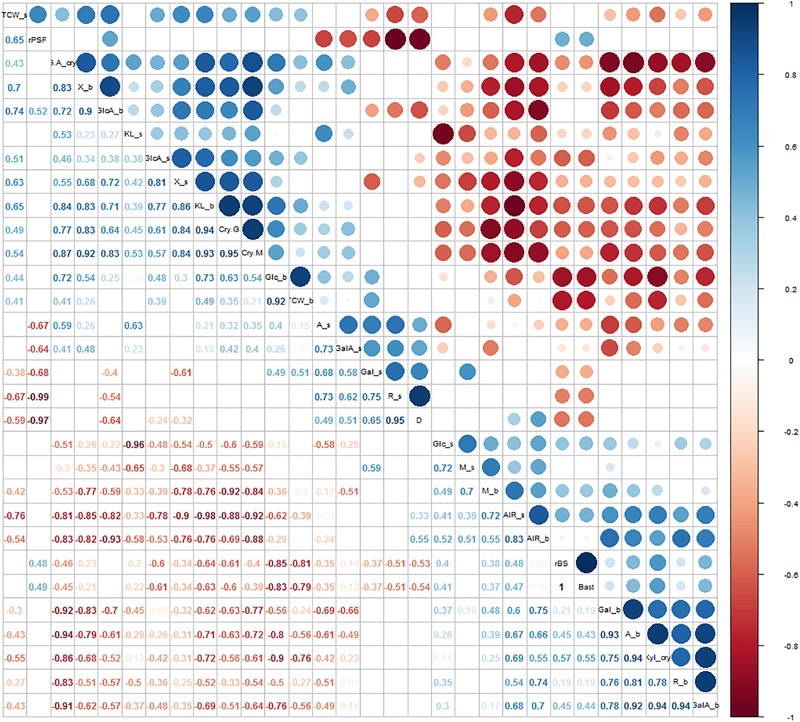
Correlation analysis between morphological parameters and cell wall traits. Significant correlations were set at a confidence level of 0.95 and blank cells represent no significant correlations. **A_s** = arabinose from the stem; **Gal_s** = galactose from the stem; **GalA_s** = galacturonic acid from the stem; **Glc_s** = glucose from the stem; **GlcA_s** = glucuronic acid from the stem; **M_s** = mannose from the stem; **R_s** = rhamnose from the stem; **X_s** = xylose from the stem; **KL_s** = Klason lignin from the stem; **TCW_s** = total cell wall components from the stem; **AIR_s** = alcohol insoluble solids fraction from the stem; **A_b** = arabinose from the bast; **Gal_b** = galactose from the bast; **GalA_b** = galacturonic acid from the bast; **Glc_b** = glucose from the bast; **GlcA_b** = glucuronic acid from the bast; **M_b** = mannose from the bast; **R_b** = rhamnose from the bast; **X_b** = xylose from the bast; **KL_b** = Klason lignin from the bast; **TCW_b** = total cell wall components from the bast; **AIR_b** = alcohol insoluble solids fraction from the bast; **CryG** = percentage of crystalline cellulose; **CryM** = percentage of crystalline mannan; **GA_cry** = percentage of galacturonic acid in the crystalline cell wall fraction; **Xyl_cry** = percentage of xylose in the crystalline cell wall fraction; **D** = diameter; **rBS** = ratio between bast and WHC; **Bast** = area of bast; **rPSF** = ratio between primary bast and secondary bast fiber.

**FIGURE 6:**
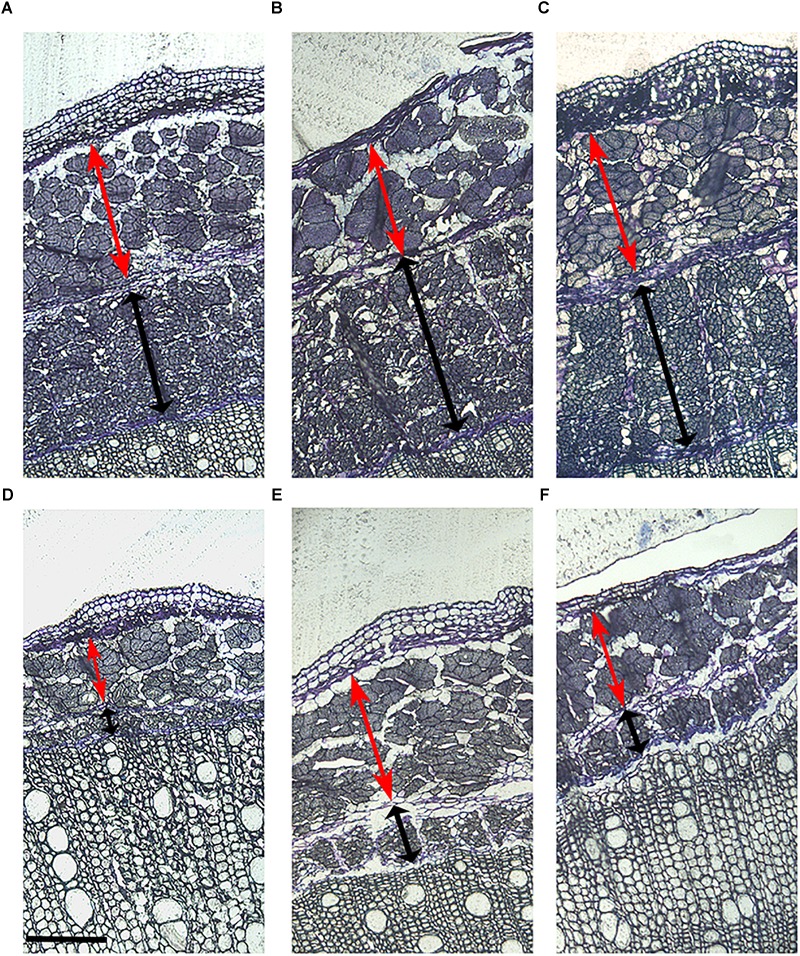
Primary and secondary bast fiber of six contrasting hemp accessions. **(A)** CRA412, **(B)** FNPC243, **(C)** WU101, **(D)** CRA410, **(E)** CRA416, and **(F)** CRA420. Red arrows indicate the primary bast fiber, black arrows indicate the secondary bast fiber and scale bars are equivalent to 200 μm.

The morphological structure of the stem was studied in the six hemp accessions to evaluate the organization of the different fibers in the stem and their relationship with the biochemical composition. The morphological study was based on the localization of the polysaccharides and lignin in the stem. CRA412, FNPC243, and WU101 stems were the ones with the largest diameters while CRA410, CRA416, and CRA420 stems were thinner (Supplementary Figure 2). WU101 stem was the thickest with the lowest proportion of bast area while CRA416 stem was thin with the largest proportion of bast area, as shown in Figure 4, Table 5, and Supplementary Figure 3. The ratio bast/WHC and the area of bast fiber were negatively correlated to the stem diameter (*r* = -0.53 and *r* = -0.54, respectively) (Figure 5). Analysis of the ratio between the primary bast fiber and the secondary bast fiber in the various hemp accessions showed that this ratio was negatively correlated to the stem diameter (*r* = -0.97), meaning that the accessions with thicker stems showed larger proportion of secondary bast fibers (Figure 5, 6 and Table 5). Altogether, significant differences were found between stem morphology of distinct accessions.

**Table 5:** Stem morphology characteristics of six contrasting hemp accessions.

Trait	CRA410	CRA412	CRA416	CRA420	FNPC243	WU101	Sign. level
Stem diameter (cm)	0.68 ± 0.029 (4.22)	1.82 ± 0.076 (4.2)	0.9 ± 0.1 (11.11)	0.9 ± 0 (0)	1.73 ± 0.061 (3.33)	2.1 ± 0.1 (4.76)	^∗∗∗^
Ratio Bast/WHC	0.197 ± 0.005 (2.46)	0.218 ± 0.023 (10.48)	0.274 ± 0.024 (8.86)	0.239 ± 0.013 (5.48)	0.21 ± 0.012 (5.73)	0.161 ± 0.013 (8.02)	^∗∗∗^
Bast area (%)	16.45 ± 0.338 (2.06)	17.86 ± 1.523 (8.53)	21.51 ± 1.504 (6.99)	19.28 ± 0.85 (4.41)	17.32 ± 0.823 (4.75)	13.86 ± 0.956 (6.90)	^∗∗∗^
Ratio Primary/secondary bast	3.15 ± 0.53 (16.95)	1.17 ± 0.09 (8.11)	2.58 ± 0.19 (7.43)	2.62 ± 0.38 (14.62)	1.19 ± 0.33 (27.58)	0.84 ± 0.22 (26.19)	^∗∗∗^

*Values indicated correspond to mean ± standard deviation. Percentages of coefficient of variation (CV%) between technical replicates are shown between brackets. ^∗∗∗^*p* < 0.001.*

Relationships between cell wall components and stem morphological measurements were investigated and high significant correlations were identified (Figure 5). These included negative correlations between the content of arabinose and galactose in the stem and the ratio between primary bast and secondary bast fibers, the area of bast and the ratio bast/WHC. Positive correlations were observed between the content of arabinose and galactose in the stem and stem diameter. By contrast, content of arabinose, galactose, galacturonic acid, and rhamnose in the bast fiber was positively correlated to the area of bast and the ratio bast/WHC. The content of xylose, glucuronic acid, and Klason lignin in both stem and bast fiber and the percentage of crystalline structures from the bast were negatively correlated to the area of bast and the ratio bast/WHC. Furthermore, the content of glucose in the stem correlated positively with the area of bast and the ratio bast/WHC while the content of glucose in the bast fiber correlated negatively with those morphological measurements. Finally, the content of mannose in the bast fiber showed a positive correlation with the area of bast and the ratio bast/WHC.

### The Protocols Are Highly Repeatable

Most traits analyzed showed small standard deviations and coefficients of variation between technical replicates (Figure 3, Tables 3–6, Supplementary Figures 2, 3, and Supplementary Table 2) indicating that the methods used are highly repeatable. Only components that were present in low contents in certain tissues such as xylose, glucuronic acid, and lignin content (KL) in the bast fiber (Crônier et al., 2005) showed larger coefficients of variation in some accessions. Generally, the biochemical variation between technical replicates were slightly larger in the stem than in the bast fiber (Figure 3 and Supplementary Table 2).

**Table 6:** Radium (in mm) of the primary and secondary bast fiber, woody hemp core (WHC), and lumen from the cross-sections of hemp stems.

Radium (mm)	Cross section	Technical replicate	CRA410		CRA412		CRA416		CRA420		FNPC243		WU101	
Primary Bast	1	1	109.24	(20.01)	229.17	(16.99)	263.78	(20.36)	231.00	(13.99)	256.58	(23.63)	211.54	(32.53)
		2	159.66		204.17		181.10		194.53		276.32		142.31	
		3	142.86		270.83		177.17		234.04		230.26		196.15	
	2	1	158.91		195.83		213.38		185.61		222.51		273.03	
		2	135.66		241.67		156.05		174.24		240.84		322.37	
		3	139.53		250.00		219.75		159.09		251.31		161.18	
	3	1	90.00		229.17		267.86		213.59		106.51		116.07	
		2	180.00		337.50		187.50		189.32		186.39		285.71	
		3	167.50		245.83		160.71		165.05		186.39		205.36	

Secondary bast	1	1	33.61	(38.09)	241.67	(17.15)	70.87	(37.21)	94.22	(27.43)	177.63	(11.54)	261.54	(9.9)
		2	46.22		133.33		74.80		97.26		220.39		276.92	
		3	84.03		200.00		90.55		94.22		167.76		261.54	
	2	1	31.01		191.67		121.02		71.97		178.01		220.39	
		2	31.01		241.67		79.62		64.39		164.92		243.42	
		3	58.14		225.00		54.14		75.76		180.63		240.13	
	3	1	35		250.00		56.55		97.09		177.51		285.71	
		2	57.5		212.50		44.64		48.54		224.85		245.54	
		3	40		241.67		35.71		43.69		192.31		303.57	

WHC	1	1	1025.21	(7.09)	2791.67	(7.11)	881.89	(14.64)	1079.03	(13.8)	2220.39	(11.71)	3934.62	(8.12)
		2	873.95		2641.67		862.20		1091.19		2167.76		4330.77	
		3	1067.23		2541.67		870.08		1209.73		2648.03		3880.77	
	2	1	883.72		2929.17		1248.41		1007.58		2099.48		3776.32	
		2	984.50		2820.83		1063.69		814.39		2198.95		3657.89	
		3	1003.88		2587.50		894.90		799.24		2651.83		3187.50	
	3	1	897.50		2570.83		952.38		1053.40		2026.63		3834.82	
		2	992.50		2466.67		1154.76		883.50		1866.86		3700.89	
		3	1010.00		2333.33		1148.81		975.73		2292.90		3589.29	

Lumen	1	1	357.14	(23.18)	0.0	(150.78)	358.27	(26.04)	395.14	(32.97)	305.92	(40.29)	361.54	(16.84)
		2	386.55		0.0		322.83		395.14		171.05		415.38	
		3	382.35		0.0		385.83		370.82		434.21		330.77	
	2	1	182.17		0.0		286.62		174.24		316.75		384.87	
		2	267.44		0.0		455.41		628.79		253.93		421.05	
		3	403.10		0.0		340.76		250.00		204.19		299.34	
	3	1	357.50		191.7		187.50		383.50		443.79		433.04	
		2	257.50		187.5		241.07		388.35		390.53		459.82	
		3	295.00		225.0		252.98		451.46		109.47		522.32	

*Percentages of coefficient of variation (CV%) between the replicates are shown between brackets.*

### The Throughput of the Protocols for Biochemical and Morphological Analysis of Hemp Stems Was Upgraded

The throughput of the protocols for hemp cell wall biochemical characterization was improved by increasing the amount of cell wall extracted in a single round. The starting amount of biomass was scaled up from 10 to 50 mg of the original protocol (Pettolino et al., 2012) to 1 g. The duration of the AIR preparation remained the same after this modification. Approximately 16 h were necessary to extract the cell wall. Between 3 and 4 h were necessary for each part of the protocol: cell wall extract, α-amylase digestion and drying of the pellets after extraction and digestion. A single protocol for monosaccharide characterization and Klason lignin analysis also improved the throughput of the biochemical characterization of hemp cell walls. This protocol also enabled the simultaneous analysis of more samples, 60 samples per run.

The original procedure for the stem morphology (Technovit^®^ 7100 Kit -Heraeus Kulzer- procedure) required 7 days to fixate, infiltrate, and embed the samples. The throughput of the protocol developed for analysis of hemp stem morphology was improved by reducing the duration of the procedure to 4 days.

## Discussion

The growing need toward a Circular Bioeconomy requires crops that stand for alternative sustainable solutions. Such requirements increase the need for phenotyping methods suitable for breeding programs for fiber crops, such as hemp. In the present study, we optimized the suitability, repeatability and the throughput of five methods to study the biochemical composition and the morphology of hemp stems.

### Large Diversity in Biochemical Composition and Morphology of Hemp Stem

Distinct tissues in the stems of hemp differ in cell wall content and composition, which supports previous findings (Bonatti et al., 2004; Crônier et al., 2005; van den Broeck et al., 2008). The bast fiber showed larger cell wall content than the stem (Figure 3), which is in accordance with differences in cell wall thickness between the bast and the WHC. The thicker walls from phloematic cells compared to xylem cells might explain the differences in cell wall content between the bast and the stem (Toonen et al., 2004; Hughes, 2012). Differences in biochemical composition between tissues are essentially in agreement with previous reports. Hemp stems had a higher content of xylose, glucuronic acid and lignin than the bast, which could be explained by the larger contents of xylan and lignin in the WHC than in the bast fiber (Bonatti et al., 2004; van den Broeck et al., 2008). By contrast, bast fiber had larger content of glucose and mannose owed to the larger content of cellulose and mannan in the bast than in the WHC (Crônier et al., 2005). Furthermore, significant differences of galacturonic acid, the main monosaccharide from pectin (Willats et al., 2001), were found between the bast fiber and the stem. Pectin in the stems of hemp is largely found in the bast fiber and in the middle lamella of the vascular cambium between the bast and the WHC (Crônier et al., 2005). An explanation for the large differences between tissues could be due to partial retting of the stems. Retting is a post-harvest treatment of the stems that facilitates the separation of the bast and the WHC. This post-harvest processing mostly degrades the pectin from the middle lamella of the vascular cambium facilitating this way the detach of bast fibers (Liu et al., 2015). Despite the differences between tissues, the content of monosaccharides composing pectin from the bast fiber are comparable to those described in previous reports (Crônier et al., 2005).

Cell wall composition of both stem and bast fiber between the six hemp accessions is largely diverse (Figure 3), which is in line with the large diversity in fiber quality, morphology, and biochemical composition of hemp stems described in previous reports (Meijer, 1994; Meijer and Keizer, 1996; Struik et al., 2000; Toonen et al., 2004; Mankowska et al., 2006; Amaducci et al., 2008; Jankauskiene et al., 2015; Müssig and Amaducci, 2018; Wang et al., 2018). Furthermore, the phenotypic variation of cell wall components was different in the bast and in the stem, suggesting that cell wall composition in different stem tissues is regulated differently (van den Broeck et al., 2008). Xylose and mannose play important roles in the mechanical properties of the fibers (Schönberg et al., 2001; Sorieul et al., 2016), and thus accessions that differ in the composition of these polysaccharides, will most likely have different functionalities. For instance, accessions with larger xylan content could produce extended or stronger polysaccharide matrixes in the WHC enhancing the recalcitrance of the stem (Torres et al., 2013).

Crystalline polysaccharides are highly abundant in the bast fiber of hemp supporting previous findings (Table 3) (Millane and Hendrixson, 1994; Crônier et al., 2005). In addition, the large diversity in crystalline cellulose and mannan between the six hemp accessions could also influence the mechanical properties of hemp fiber, as previous reports suggested that crystallinity might affect fiber strength (Bourmaud et al., 2013; Marrot et al., 2013). Remarkably, a fraction of xylose, galacturonic acid and rhamnose were detected in the crystalline cell wall fraction, suggesting that crystalline polysaccharides might protect a fraction of xylan and pectin, which is in line with previous reports using immunohistochemistry (Gorshkova et al., 2010; Chernova et al., 2018). The large proportion of galacturonic acid found entrapped by crystalline polysaccharides (Table 4) could indicate the existence of a pectic polymer with a rhamnogalacturonan I backbone (galacturonic acid and rhamnose) in the G-layer of the bast fiber, as suggested by Chernova et al. (2018). Furthermore, the large variation of galacturonic acid content in this fraction between the six hemp accessions (Table 4) could play a role in the regulation of the crystallinity, thus affecting the properties of the crystalline polysaccharides.

The organization of the different fibers in the stem is diverse between the six hemp accessions (Figure 4, 6) and is highly correlated to the biochemical composition of the stem (Figure 5), as expected from previous studies (Bonatti et al., 2004; Crônier et al., 2005; van den Broeck et al., 2008; Behr et al., 2016). The bast area and the ratio bast/WHC were positively correlated to the content of glucose and negatively correlated to the content of xylose and lignin, which support previous findings of the cell wall composition from the bast and the WHC. Bast fibers are characterized by large content of cellulose while WHC has large content of xylan and lignin (Bonatti et al., 2004; van den Broeck et al., 2008). The ratio between primary bast and secondary bast fibers was negatively correlated to the contents of arabinose and galactose. Arabinose and galactose are the monosaccharides of the side-chain substitutions of the pectin-type RGI (Willats et al., 2001). The relationship suggests a larger content of pectin substitutions in the secondary bast fiber than in the primary bast fiber. The larger content of these substitutions in the secondary bast fiber suggests stronger polysaccharide matrixes in the cell walls, due to larger amount of cross-links between the side-chains of RGI (Willats et al., 2001). As a consequence, the large content of pectin substitutions might partially explain the large stiffness associated to the secondary bast fibers. Additionally, pectin hampers post-harvest processing of the fibers, such as decortication or scutching (Müssig and Martens, 2003). Therefore, this relationship suggest positive implications for the fiber quality of hemp because large primary bast fibers and low pectin content are highly appreciated, especially for the textile industry (Müssig and Martens, 2003; Chernova and Gorshkova, 2007; Amaducci et al., 2015). Lower content of pectin substitutions in the stem may shorten the retting time of the stems. As a result, the bast fibers are less damaged, which is consistent with previous studies (Liu et al., 2015). Furthermore, the relationships between some stem morphological measurements and the biochemical composition of the bast fibers revealed remarkable results. The bast area and the ratio bast/WHC negatively correlate to the content of glucose and to the percentage of crystalline polysaccharides from the bast while they positively correlated to the content of mannose and to the monosaccharides composing pectin in the bast. It seems plausible that those relationships refer specially to an increase of the middle lamella from the bast fiber with the increase of bast area or ratio bast/WHC. Therefore, the increase of bast area might be related to an expansion of the bast in surface owed to the increase of middle lamella instead of the increase of total cell wall or the increase of the main components from the bast, such as cellulose. These results are beneficial for the breeding for fiber quality, as discussed in previous reports, there is a positive relationship between the middle lamella from the bast fiber bundles and the mechanical properties of the bast fibers (Fuentes et al., 2017).

### Highly Repeatable and Upgraded High-Throughput Methods for Hemp Breeding Programs

The methods developed and optimized to study the biochemical composition and the morphology of hemp stems show large repeatability as it can be concluded from the small differences between technical replicates (Figure 3 and Tables 3–6), suggesting the suitability of the upgraded methods for the phenotyping and breeding for hemp fiber quality. High quality biochemical and morphological data is essential to study the phenotypic variation between accessions and to be able to identify the genetic architecture underlying quantitative complex traits such as fiber quality (Huang and Han, 2014). Cell wall extraction using the high-throughput methods currently available, such as NDFs (Goering and Van Soest, 1970) are based on aqueous solvents. Those methods are not suitable to extract cell walls comprising important water-soluble components, such as some pectin polysaccharides (Pettolino et al., 2012). The contents of monosaccharides composing pectin (Figure 2, 3) from the bast fiber in the present study were comparable to those described in previous reports that used long sequential extraction procedures (Crônier et al., 2005). The alcoholic phobicity properties of the polysaccharides, including pectin, and lignin (Fry, 2003; Huffman and Caballero, 2003; Pettolino et al., 2012; Chen, 2014) can explain the quantification of the almost complete cell wall mass using the optimized methods based on alcohol-based solvents. In addition, the heterogeneous morphology of hemp stems and the large abundance of crystalline polysaccharides in the hemp fibers hinder the complete study of the cell wall composition, which is in line with previous reports (Crônier et al., 2005; Pettolino et al., 2012; Pu et al., 2013). The large repeatability of the monosaccharides and Klason lignin contents between the technical replicates and the almost complete cell wall mass (Figure 3) suggest that the optimized two-step sulfuric acid hydrolysis is nearly complete and without degradation of released sugars, as furfural and hydroxy-methyl furfural (HMF) were not detectable (data not shown). The modifications of the hydrolysis protocol may allow a better disruption of the cell walls which in turn allow a deeper accessibility of the acid through the cell wall structures allowing a harsher acid performance, which is supported by previous studies (Zhao et al., 2006). Thus, the methods presented in this report combine the high-throughput with the accuracy of the best methods available for cell wall characterization in dicot plants adapted to hemp limitations. These two features are key for the phenotyping of a large number of plants but maintaining the quality of the phenotypic data needed for quantitative approaches (Huang and Han, 2014).

Altogether, the accuracy of the methods developed for cell wall composition generated high quality data that can be used to develop prediction models with near-infrared spectroscopy (NIRS) (Toonen et al., 2004), and thus increase even further the throughput of hemp cell wall biochemical analysis and phenotyping of large number of samples for breeding (Huang and Han, 2014). Notwithstanding, our methods already have the potential to phenotype large number of accessions in a relatively short period of time. Consequently, the methods can already be used to phenotype plants for association studies for fiber quality of hemp.

## Conclusion

Repeatable and high-throughput phenotyping methods to study the genetic diversity of fiber hemp are key before we can breed for hemp varieties with better biomass composition. In the present study, we have developed and optimized five methods to study the phenotypic variation of cell wall composition and stem morphology of hemp. The methods revealed to be repeatable and sensitive to discriminate differential composition between hemp accessions, and thus suitable for characterizing the variation in fiber quality of this not extensively breed crop. In addition, the throughput of the methods was significantly upgraded to enable the exploration of the fiber quality and will contribute in the development of breeding tools. The development of such phenotypic methods will have a valuable impact on the study of hemp and will accelerate the breeding for hemp varieties with better quality of fiber.

## Author Contributions

JP designed and performed the experiments, analyzed the data, and wrote the manuscript. AG helped designing and performing the experiments, and revised the manuscript. AD optimized the experiments and data analysis, and revised the manuscript. LT coordinated and supervised this study, experimental strategy, and discussion of the outcomes, and revised the manuscript.

## Conflict of Interest Statement

The authors declare that the research was conducted in the absence of any commercial or financial relationships that could be construed as a potential conflict of interest.
