# Supplementary material for: Phenotypic Variation of Cell Wall Composition and Stem Morphology in Hemp (Cannabis sativa L.): Optimization of Methods
Source: Front Plant Sci. 2019 Jul 25;10:959. doi: 10.3389/fpls.2019.00959 (PMC6671528; doi:10.3389/fpls.2019.00959)
Supplement: Supplementary file 1 [file Table_1.docx]

**Supplementary Table 1.** Concentration of each monosaccharide in the set of carbohydrate recovery standards (SRS).

| Monosaccharide | Concentration [mg/ml] |
| --- | --- |
| Rhamnose | 0.016 |
| Arabionse | 0.18 |
| Galactose | 0.43 |
| Glucose | 0.8 |
| Xylose | 0.43 |
| Galacturonic acid | 0.16 |
| Glucuronic acid | 0.011 |
| Mannose | 0.07 |

**Supplementary Table 2.** Content of monosaccharides and Klason lignin and percentage of AIR fraction (total cell wall) in the stem and in the bast fibre of six contrasting hemp accession.

| Trait | Description | CRA410 | | CRA412 | | CRA416 | | CRA420 | | FNPC243 | | WU101 | | Differences accessions | | Differences tissues |
| --- | --- | --- | --- | --- | --- | --- | --- | --- | --- | --- | --- | --- | --- | --- | --- | --- |
|  |  | Stem | Bast | Stem | Bast | Stem | Bast | Stem | Bast | Stem | Bast | Stem | Bast | Stem | Bast |  |
| Ara%AIR | Arabinose | 0.56 ± 0.025 (4.54) | 0.48 ± 0.003 (0.69) | 0.58 ± 0.038 (6.68) | 0.74 ± 0.019 (2.70) | 0.57 ± 0.011 (1.9) | 0.86 ± 0.035 (4.02) | 0.55 ± 0.088 (15.96) | 0.71 ± 0.006 (0.78) | 0.68 ± 0.042 (6.19) | 0.36 ± 0.009 (2.61) | 0.61 ± 0.056 (9.07) | 0.7 ± 0.019 (2.72) | * | *** | *** |
| Gal%AIR | Galactose | 1.11 ± 0.068 (6.14) | 1.69 ± 0.03 (1.78) | 1.16 ± 0.043 (3.70) | 1.97 ± 0.082 (4.18) | 1.1 ± 0.024 (2.18) | 2.08 ± 0.014 (0.68) | 1.18 ± 0.05 (4.26) | 2.1 ± 0.01 (0.5) | 1.21 ± 0.059 (4.92) | 1.36 ± 0.03 (2.23) | 1.17 ± 0.027 (2.28) | 2.09 ± 0.034 (1.64) | * | *** | *** |
| GalA%AIR | Galacturonic acid | 4.6 ± 0.252 (5.48) | 2.47 ± 0.04 (1.61) | 5.05 ± 0.266 (5.27) | 4.73 ± 0.039 (0.83) | 4.59 ± 0.167 (3.65) | 4.4 ± 0.131 (2.98) | 4.56 ± 0.572 (12.54) | 3.75 ± 0.007 (0.18) | 5.18 ± 0.153 (2.94) | 2.35 ± 0.033 (1.4) | 4.65 ± 0.459 (9.88) | 3.54 ± 0.111 (3.12) | n.s. | *** | *** |
| Glc%AIR | Glucose | 53.89 ± 1.18 (2.19) | 78.67 ± 0.53 (0.67) | 55.22 ± 1.22 (2.21) | 75.5 ± 0.1 (0.13) | 53.44 ± 2.33 (4.36) | 71.16 ± 0.61 (0.86) | 58.65 ± 1.95 (3.32) | 75.9 ± 1.34 (1.77) | 52.1 ± 0.71 (1.35) | 78.29 ± 0.95 (1.21) | 52.36 ± 2.08 (3.98) | 77.91 ± 1.53 (1.97) | ** | *** | *** |
| GlcA%AIR | Glucuronic acid | 0.603 ± 0.009 (1.52) | 0.178 ± 0.012 (6.98) | 0.348 ± 0.005 (1.4) | 0.095 ± 0.033 (34.78) | 0.389 ± 0.027 (6.87) | 0.102 ± 0.026 (25.15) | 0.335 ± 0.032 (9.46) | 0.068 ± 0.019 (27.36) | 0.363 ± 0.012 (3.27) | 0.131 ± 0.031 (23.45) | 0.521 ± 0.015 (2.91) | 0.045 ± 0.024 (53.5) | *** | *** | *** |
| Man%AIR | Mannose | 2.03 ± 0.109 (5.35) | 5.65 ± 0.175 (3.1) | 1.97 ± 0.054 (2.72) | 5.49 ± 0.142 (2.58) | 1.76 ± 0.065 (3.71) | 7.57 ± 0.111 (1.47) | 3.27 ± 0.135 (4.15) | 8.86 ± 0.019 (0.21) | 2.32 ± 0.039 (1.67) | 6.55 ± 0.086 (1.31) | 2.35 ± 0.108 (4.61) | 7.16 ± 0.065 (0.91) | *** | *** | *** |
| Rha%AIR | Rhamnose | 0.78 ± 0.035 (4.41) | 0.688 ± 0.003 (0.4) | 0.84 ± 0.026 (3.05) | 0.939 ± 0.023 (2.45) | 0.80 ± 0.03 (3.69) | 0.806 ± 0.025 (3.07) | 0.81 ± 0.041 (5.03) | 0.803 ± 0.01 (1.29) | 0.85 ± 0.048 (5.61) | 0.639 ± 0.008 (1.33) | 0.86 ± 0.03 (3.48) | 0.799 ± 0.017 (2.18) | n.s. | *** | *** |
| Xyl%AIR | Xylose | 17.43 ± 0.44 (2.52) | 2.72 ± 0.102 (3.75) | 14.17 ± 0.473 (3.34) | 1.93 ± 0.260 (13.23) | 13.86 ± 0.846 (6.11) | 1.18 ± 0.176 (14.92) | 11.19 ± 0.852 (7.61) | 1.09 ± 0.322 (29.44) | 13.37 ± 0.235 (1.76) | 2.38 ± 0.092 (3.88) | 13.96 ± 0.076 (0.54) | 1.06 ± 0.148 (14) | *** | *** | *** |
| KL%AIR | Klasson lignin | 14.18 ± 0.45 (3.19) | 2.6 ± 0.13 (5.15) | 13.26 ± 0.54 (4.11) | 1.98 ± 0.3 (15.34) | 14.86 ± 0.67 (4.48) | 1.68 ± 0.2 (11.73) | 12.2 ± 0.96 (7.85) | 1.61 ± 0.14 (8.68) | 15.1 ± 0.34 (2.24) | 2.16 ± 0.38 (17.38) | 14.73 ± 0.33 (2.24) | 2.07 ± 0.13 (6.11) | *** | ** | *** |
| Total cell wall components | Sum of monosaccahrides and lignin | 95.19 ± 2.01 (2.11) | 95.15 ± 0.41 (0.43) | 92.58 ± 2.44 (2.63) | 93.38 ± 0.79 (0.85) | 91.37 ± 3.88 (4.25) | 89.85 ± 0.35 (0.39) | 92.73 ± 0.4 (0.43) | 94.89 ± 1.01 (1.07) | 91.94 ± 0.24 (0.27) | 94.22 ± 1.12 (1.19) | 91.21 ± 1.45 (1.59) | 95.37 ± 1.79 (1.88) | n.s. | *** | * |
| AIR%DM | Alcohol insoluble solids fraction | 89.9 ± 0.18 (0.2) | 96.42 ± 1.57 (1.62) | 92.26 ± 0.98 (1.06) | 98.69 ± 0.31 (0.31) | 92.84 ± 1.05 (1.13) | 97.82 ± 0.14 (0.15) | 93.2 ± 1.59 (1.7) | 99.19 ± 0.42 (0.42) | 91.69 ± 1.43 (1.56) | 97.13 ± 0.54 (0.55) | 92.2 ± 1.19 (1.19) | 98.74 ± 1.2 (1.22) | * | * | *** |

The values presented correspond to the means ± standard deviation. Percentages of coefficient of variation (CV%) between technical replicates are shown between brackets. Significant levels: *, **, *** and n.s. correspond to significant differences at *p* < 0.1, *p* < 0.01, *p* < 0.001 and no-significant respectively.


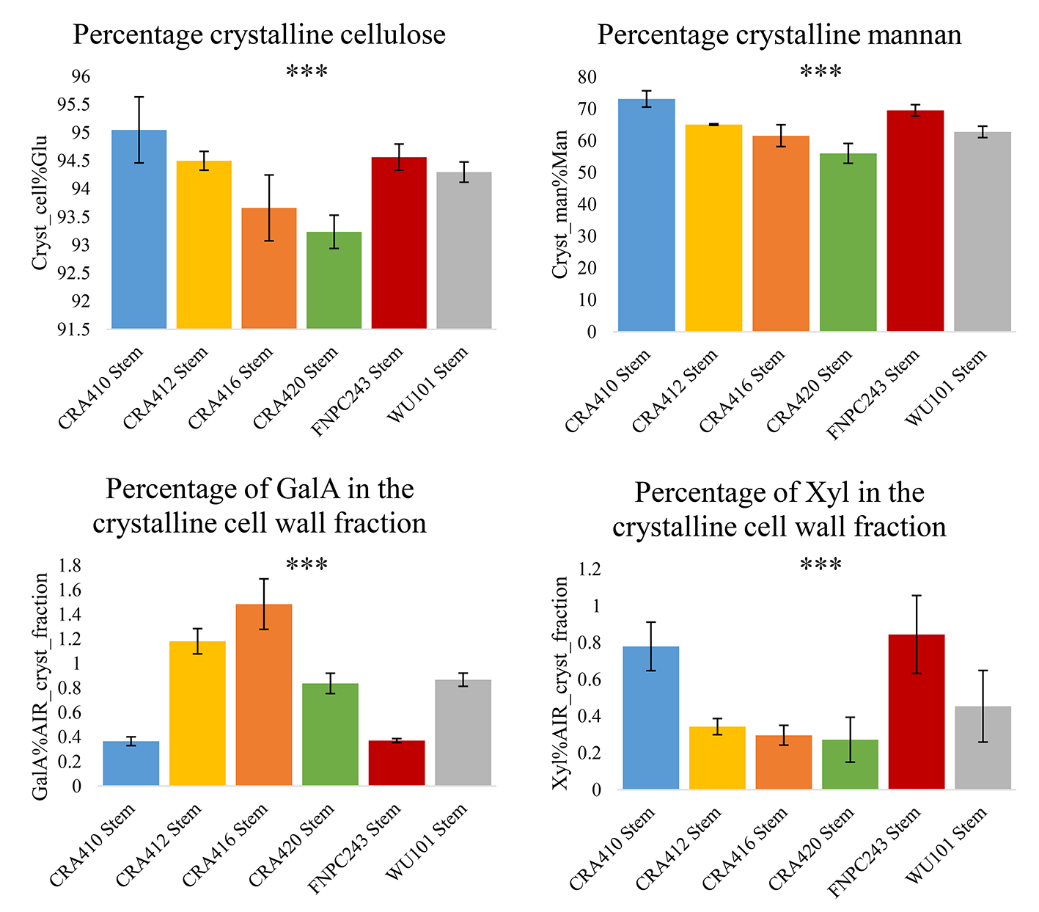


**Supplementary Figure 1.** Percentage of crystalline cellulose (**A**) and mannan (**B**) in bast fibre of six contrasting hemp accessions. Content of galacturonic acid (GalA) (**C**) and xylose (Xyl) (**D**) detected in the crystalline fraction of the fix hemp accessions. The columns represent the means and the bar of each column represents the standard deviation. Significant levels: **, *** correspond to significant differences at *p* < 0.01 and *p* < 0.001, respectively.


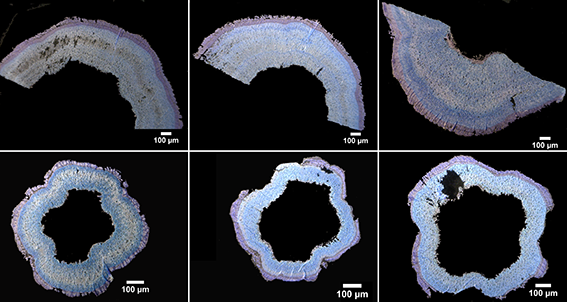


**Supplementary Figure 2.** Stem morphology of six contrasting hemp accessions. **A**) CRA412, (**B**) FNPC243, (**C**) WU101, (**D**) CRA410, (**E**) CRA416 and (**F**) CRA420.


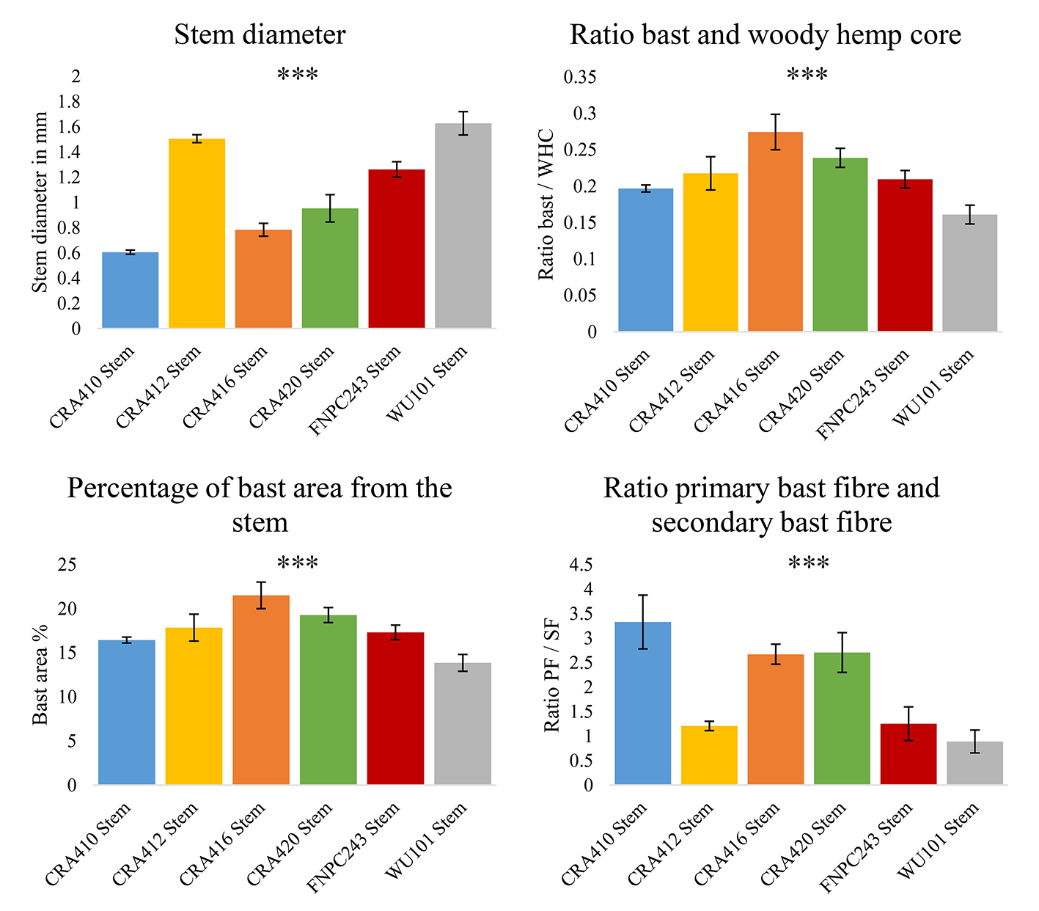


**Supplementary Figure 3.** Stem morphology characteristics of six contrasting hemp accessions: stem diameter (**A**) ratio bast and woody hemp core (**B**), percentage of bast area from the stem (**C**) and ratio primary bast fibre and secondary bast fibre (**D**). The columns represent the means and the bar of each column represents the standard deviation. Significant levels: **, *** correspond to significant differences at *p* < 0.01 and *p* < 0.001, respectively.
